# Health professionals’ experiences with the implementation of a digital medication dispenser in home care services – a qualitative study

**DOI:** 10.1186/s12913-020-05191-9

**Published:** 2020-04-16

**Authors:** Hanne H. Kleiven, Birgitte Ljunggren, Marit Solbjør

**Affiliations:** 1 Department of Social Science, QMUC, Thrond Nergaards veg 7, N-7044 Trondheim, Norway; 2 Faculty of Medicine and Health Science, Department of Public Health and Nursing, NTNU, 7491 Trondheim, Norway

**Keywords:** Welfare technology, Implementation, Digitalization, Home care service, Science and technology studies, Domestication, Script, Scandinavia

## Abstract

**Background:**

Implementing digital technology in home care services challenges care arrangements built on face-to-face encounters. Digital welfare technology has been suggested as a solution to increasing demands on health care services from an ageing population. Medication delivery is a major task for home care services, and digital medication devices could lessen the need for resources. But technology has scripts based on how designers picture its use, and these might not fit with users’ needs and practices. New technology must go through processes of domestication among its users. In the present study, we investigate how health professionals experienced the implementation of a digital medication dispenser into home care services in Norway.

**Methods:**

This was a qualitative interview study with 26 health professionals from home care services in five municipalities.

**Results:**

All five municipalities had implemented a digital medication dispenser in home care services. Prior to the introduction of the dispenser, medication practices had been based on home visits. The safety of medication practices was the main concern of health professionals who had to negotiate the technological script in order to make it work in a new care arrangement. Rationalities of effectiveness collided with rationalities of care, symbolized by warm hands. Professionals who had been used to working independently became dependent on technical support. Being unfamiliar with the new medication arrangement led to resistance towards the digital dispenser, but more direct experiences changed the focus from technology to new care arrangements. Negotiating practical and organizational arrangements led health professionals to trust the digital medication dispenser to contribute to safe and good care for service users.

**Conclusions:**

Implementing digital technology in home care services must be informed by previous practices in the field, especially when it concerns safety for patients. Through processes of domestication, health professionals negotiate technological scripts to make them fit professional ideals and practices. Policymakers and managers must address questions of care arrangements and individualized adaptions to patients’ needs in order to receive support from health professionals when implementing digital technology in home care services.

## Introduction

Welfare technology, which is digital assistive technology for use in relation to health care services, has been suggested as a solution to future challenges in the Scandinavian countries [1, 2]. Ongoing demographic change causes higher numbers of old people in need of health care services, leading to unsustainable costs for welfare states. Moreover, demographic change lead to a decrease in the working population, including the number of health care professionals. Implementation of welfare technology is one way to meet these challenges [3]. Welfare technology is expected to decrease costs, supplement or replace staff, secure quality of care, and provide help and empowerment to users of health care services. In Norway, the government launched a national policy to encourage innovation and development of welfare technology [4].

In Scandinavia, municipal home care services are expected to benefit from welfare technology, since it could potentially increase the time that elderly users could live at home and decrease the number of daily visits from home care services to each individual user [5]. Home care services in Norway are based on home visits to each service user, up to several times a day if needed. With potentially long distances to drive to users’ homes due to the scattered population in many municipalities, home visits are time-consuming. Through welfare technology, care can be managed from a distance, i.e. by screen or through an alarm central [6, 7]. For welfare technology to allow elderly individuals to stay at home, digital technology must be implemented into patients’ homes and become part of the services of home care services.

For home care services, the administration of medication often ties up much of the daily schedules of nurses [8]. The administration of medication demands quality control and ensuring safety. It is closely connected to other nursing practices such as observations and face to face interactions. Introducing a digital medication device could and should lead to fewer visits and thereby less contact between service users and health professionals. Historically, home care services were initiated as a substitute or supplement to familial care [9]. Older home care service users are often described as potentially experiencing loneliness and in need of human interaction with health care providers. Thus, introducing digital technology for home care services could face resistance if care is seen as “warm” while technology is seen as “cold”, suggesting a dehumanization of care [10].

Health care professionals are key to the implementation of welfare technology [11]. Previous research has identified health care professionals as sceptical to new technology in the care for the elderly due to fear of being substituted, experiencing technical barriers, or seeing it as an extra task in their already overfilled workload [1, 12]. Some professionals worry that digital medication technology will alter their relationship with patients [5]. Organizational, cultural, technological, and ethical forms of resistance towards the implementation of welfare technology in municipal eldercare organizations have been found in studies from Norway [13] and Sweden [1]. But these studies lack information on whether these findings also apply to home care services. Despite resistance, health policy in Scandinavia continues to call for more digital solutions in municipal health care services. In the present study, we investigate how health professionals experienced the implementation of a digital medication dispenser into home care services in five Norwegian municipalities.

### Technological implementation in a care context

The implementation of new technology in home care services aim to contribute to care for individuals living in their homes. But how technology is used is dependent on context, and the introduction of welfare technology may lead to different forms of use and various consequences in different settings. In the present study, we understand technology from the perspective of science and technology studies, which acknowledge that all technologies have scripts based on how designers picture its use, and actors’ roles and responsibilities are inscribed in the technology [14]. Inscribed users in technological scripts and images of such users are often represented by stereotypes [15], which do not always fit with how real users act. The arrangement of home care services builds on scripts of old age as passive, and welfare technology appears to be developed from the view that older people want to live at home [16]. In recent years, design processes for technology often have explicit approaches to user participation in the development of the new technology [17]. Such participatory design is meant to lead to technology which is better adapted to user needs than previous technological solutions [18]. Regardless of this development, the term script [14] is useful in exploring how technology is implemented and used in varied settings. Users of technology have active agency and are part of networks that negotiate its use, including alliances that may be constantly shifting [19]. These continuous negotiations create arrangements for care that are composed of humans, technologies and material artefacts, rules and regulations, budgets and national care plans [11]. How problems are constructed will influence how technology is met when implementing it in the care of the elderly [20].

Technological scripts can be challenged and negotiated, but there may be a strong push towards using certain technologies [21]. Pushing is particularly evident when a workplace is implementing new technology based on a top-down management decision. However, even when non-use of the new technology is difficult, users could negotiate how they use it. To obtain a real implementation of a new device, the technology must go through a process of domestication, which leads technology to take a natural place in everyday practices [22]. In domestication, users play an active role in the construction of use (practice) and meaning of technologies where doing technology is a multi-sited, multi-actor process [21]. Domestication concerns three features: the construction of practices related to a technological device (“artefact”), the construction of the meaning of the technological device, and cognitive processes related to learning of practices and meaning [21]. How users read, interpret and act towards an object may translate or re-script the programme of this object. Thus, domestication is a movement of objects into and within existing socio-technical arrangements [21]. In home care services, care is the main focus of the socio-technical arrangements through networks of professional practices. A professional arrangement for care includes actors such as patients and their families, health care professionals, management, technology providers, and user support [23]. Care arrangements describe how work practices, technologies, and individuals act together to ensure care for service users [11]. When implementing a digital medication dispenser, it would need to become a natural part of these work practices and care arrangements.

Domestication of technology in a workplace happens within already existing organizational, cultural, and professional tensions [24]. Its use is not free from normativity and morality on how technology should be used [16]. Moreover, diverse influences from practice and policy interact to produce technological identities, which shape the desirability and acceptability of the implementation of specific technology into health care services [19]. These symbolic understandings have implications for the implementation of technology into health care services. The administration of medication is a field which implies high levels of responsibility since faults could have fatal consequences. Therefore, it is important to understand the processes that take place when implementing a new digital device for medication into home care services. Such processes would include a number of actors such as service users, family members, health service managers, nurses, auxiliary nurses, technicians, and others living or working in the community. In the present study, we examine the experiences of professionals who took part in the implementation of a digital medication device in home care services.

## Methods

### Aim and study design

The aim of this study was to explore how health professionals experienced the implementation of a digital medication dispenser in home care services in Norway. The design was an explorative qualitative interview study with health personnel.

### Setting and the case of the digital medicine dispenser

The setting for this study was municipal home care services in Norway. The study was part of a larger interdisciplinary research project that combined perspectives from design and innovation, nursing, sociology, and philosophy.

Municipalities’ responsibilities for primary health care services have increased following the coordination reform which was implemented in 2012 in Norway. This has led to increasing pressure on municipal health care. The term *welfare technology* has been implemented in policy language following the White Paper [2]. Politically, the goal of utilizing welfare technology has been to increase the time citizens can live at home, thereby decreasing expenses within the health care sector. However, the actual implementation of welfare technology in municipal home services has proven slower than first assumed.

Municipalities in the present study represent innovative sites for welfare technology. These municipalities have implemented a digital medication dispenser in service users’ homes. The dispenser is filled with medication for a certain period and placed within the home of the service user. It opens at a pre-set time, providing the correct amount of medicine at each time slot. When opening, the dispenser sounds an alarm to draw its user’s attention. If the medicine is not removed within a given timeframe, the home service central receives notice, and a health care professional will call the user to explore the delay. A number of digital medication dispensers are available on the market. In the present study, the municipalities used two different dispensers.

### Recruitment and study participants

At the initiation of the study, we expected municipalities to have projects on welfare technology due to the policy launched in the white paper from 2012. However, it turned out that fewer municipalities than expected had implemented welfare technology due to delays in their working plans. We identified five Norwegian municipalities that had implemented the use of a digital medication dispenser, and all five agreed to participate in the study. The five municipalities were situated in central and southeast Norway. These included one small rural municipality with less than 5000 inhabitants, two municipalities with both urban and rural areas, and 5000–49.999 inhabitants, and two urban municipalities with more than 50.000 inhabitants.

Municipalities were approached through a high-level manager who gave consent to participate on behalf of the municipality. These managers initiated contact between the researchers and professionals who had a role in the implementation or use of the digital medication dispenser. Individual participants in the study were recruited through their leader, or by the welfare technology project leader. None of the authors knew the informants or the municipality administrations before initiating the study.

Twenty-six health professionals participated in the study. They comprise nurses, assistant nurses, occupational therapists, physiotherapists, pharmaceutical staff, and one general practitioner. Some were administrative managers, and some were project leaders in charge of the implementation of welfare technology.

### Data collection

Guided by previous research and literature, we developed a thematic interview guide with questions about experiences from the implementation process. We framed each interview addressing three major topics: “*Professional roles and cooperation”, “Innovations and change management”, and “The home, and healthcare professionals’ experiences of the technology”*. An example of the type of questions we asked is “How do you experience that the new technology has changed your workday?”. Please find the interview guide in additional file 1. We conducted 26 individual semi-structured interviews with one or two interviewers present. All interviews were done during work hours at the participants’ workplace in each municipality. On average, the interviews lasted around 45 min and were audio-recorded. Interviews were carried out as a conversation between participant and interviewer, allowing for stories and reflections from the participant, and follow up questions from the interviewer.

### Data analysis

A professional transcriber transcribed the interviews verbatim from the audio files. We used thematic analysis as described in Brinkmann and Kvale [25]. The analysis was based on the questions in the interview guide but allowing for unexpected findings through inductive coding of each interview. First author read and coded all interviews, while all authors read and coded several interviews. The research team held several meetings to discuss codes and their content, during which different interpretations were developed until consensus of interpretation was reached. By comparing codes (finding similarities) and contrasting codes (searching for negative cases), the final analytical categories related to script [14] and domestication emerged [21].

### Ethical approval

The study was approved by the Norwegian Centre for Research Data (NSD reference no. 37655). All participants signed a written form of consent after having received oral and written information about the study. Municipalities are not identified by name due to the risk of identifying individual participants, and individual participants are referred to solely by their professional title.

## Results

Several actors participated in medication practices in these home services. Prior to having the digital medication dispenser, other technological artifacts had been included in the process of providing medications to service users, such as a plastic pill dispenser, multidose with pre-packed plastic bags, or a manual medication wheel. Home care services received a list of medications from either the hospital or the patients’ general practitioner. This list was first registered in the documentation program before it was sent to the pharmacy that delivered medication to home care services. The medication came in pre-packed multidose plastic bags or in original packages which nurses had to distribute to a pill dispenser. Thus, several actors and devices were already taking part in the network necessary for ensuring correct and safe medication to home care service users before the digital dispensers were implemented. When the medication was delivered in user-ready packages, changes to the medication regime that were reported to the pharmacy would not be implemented before the next delivery. This could lead to extra work for the nurses who had to make changes to the pre-packed bags or the pill dispenser. Such changes had led to mistakes in the provision of medication.

*«So, if you are in a patient’s home, it’s the person who arrives first who takes care of the papers – bring them in, make copies. After that, a nurse must take charge to organize medication. Organize a card that is brought to the medical doctor to sign. This should be the patient’s GP. The doctor has received an epicrisis or a list of the medication from the hospital which he might sign. And then it is returned to the home care office, registered into the program for documentation, and these medicines are administered to the pill dispenser or we send a change request to the pharmacy so they can arrange the multidose bags. The change request has to be sent by telefax to the pharmacy.” (Head nurse)*

Providing medication to service users often implied making a home visit to hand out medication within preset time frames. Users who needed help with medication often required other services too. The health professional visiting the user would provide several services during each visit. Sometimes, this led to forgetting medication, if the professional was on a tight schedule, and the medications were left in the car. Thus, services had experienced mistakes with medication due to human error. However, home care services had their system with home visits and arrangements including face to face contact and control with individual medication intake.

The number of individuals and devices that were part of the network for ensuring correct and safe medication for each patient, could be defined as a *care arrangement* [11]. With the implementation of the digital device, new networks had to be formed, and care arrangements changed. As before, and following the law, nurses were the ones responsible for ensuring correct medication. Introducing a digital dispenser did not alter practices related to the administration of medication into a dispenser or to the individual home. But professional responsibility did not stop when the dispenser was placed with the user. Their concern was to ensure a safe medication regime and that service users felt safe too. While professionals were used to handling analog devices to provide medication, what was new with the digital device was that it transferred responsibility for removing the medication from the dispenser and ingesting it to the service user herself. Moreover, the device could fail due to technical problems or loosing power. Professionals had to navigate the new technology to ensure their professional standards for patient security were met within the new arrangement for care which included the digital medication dispenser.

### The digital medication dispenser as an object for professional negotiation

Decisions on implementing the digital medication dispenser were taken by leaders and managers for municipal home care services. They saw the benefits of the original script of the dispenser, which was to empower users to be responsible for their medication, employing less staff in arrangements for medication and automatizing the process of medication delivery to ensure safety and quality of services. Project managers for the implementation of the digital dispenser presented it as part of quality improvement, but those working with the dispenser among service users saw it as part of efficiency demands. The initiation of the digital medication dispenser caused colliding interests between the rationality of the managers and professional values of care, especially symbolized by “warm hands”. This was particularly evident during the initiation phase before they had experienced the new dispenser in practice.

*«I wasn’t happy about it at first. I thought that they were removing the warm hands and replacing it for a cold thing instead. If you see what I mean? That we were being replaced. But I have realized that it is beneficial, my views have changed. It was unfamiliar, a strange object. My impression is that many of us thought like I did.” (Auxiliary nurse)*

A second symbolic element was the role welfare technology had in branding the municipality as modern and «high-tech». Project managers experienced that staff was suspicious that the implementation of the dispenser was a way of branding the municipality as innovative and oriented towards new solutions. This was supported by the larger network of actors that came with the digital dispenser. Some municipalities took part in evaluations of technology and had R&D collaborations with institutions such as universities, colleges, or technology developers. Having to involve in these interactions supported the sense of being part of new policies, without providing certainty about whether this policy moved services in the right direction. For some, focusing primarily on technological artefacts rather than the service users’ needs disparaged the value of the technology.

*«I have experienced a process where managers ask with impatience: where is the cool stuff, the cool gadgets? Are you just talking? That has been a general opinion, I think, that the number of gadgets is the answer to how well we have done our job, rather than exploring the patient’s needs, so to speak.” (Occupational therapist).*

Thus, for those working directly with service users, symbols of efficiency and innovation conflicted with their professional responsibility of meeting needs, giving warm care, and securing safe delivery of medication. The digital nature of the technology contributed to uncertainty and worries, and project leaders had to demystify it. Normalization could happen through comparing the digital dispenser to other technologies that were already in use in home services but named technical aids. Digitalization did, however, raise other challenges that created new scripts for medication practices.

Though health professionals were used to manoeuvring technical aids, the digital device had functions that could not be attended by a single person. Services had to make arrangements for responding to alarms, refilling dispensers, changing batteries and performing maintenance on each device. Thus, each health professional had to take part in a larger network working together to serve the technological needs, while still serving service users’ needs. For some, the digital aspect of the dispenser led to fears of ruining it or not being able to fulfil the requirements for employing it correctly.

*«Some of us are predetermined, I think, that computers and technology are scary stuff. Here, the challenge is that people need to feel safe that they won’t ruin anything or do something wrong. But they are frightened that they will ruin the settings or even the devise, so they are afraid to touch it, like. They can’t be left with that feeling and worry about having done wrong.” (Nurse and project manager).*

Technical challenges were common and had been experienced in all five municipalities. Such experiences led to the expansion of tasks for health professionals. Sometimes, the dispenser could lock itself while medication was being installed, which led to spending time on solving it through including other professionals or calling technical support. Thus, home care professionals who were used to working independently became more dependent on other services, such as technical support in order to secure a safe medication practice. This led to higher complexity in the delivery of care, while health professionals still were the ones responsible for ensuring safe medication, and some had been frightened by experiencing errors. Therefore, they would not automatically follow the script of the dispenser which would allow for less contact between the user and home services. Leaving a digital device to the responsibility of the user was incompatible with their professional values.

*«You need to remember that technology could fail. It’s important to avoid that a GPS or the dispenser alarm becomes false security which leads us to stop paying attention because we think we’ll get the cell phone alert. Because it will fail one day. The battery may be empty without us noticing, or something else goes wrong.” (Nurse).*

Health professionals feared mistakes that could lead to harm for the service user. For the professionals, technological flaws were more than a hassle. It was about less safety and risks if the medication schedule was delayed. Experiencing insecurity about correct performance led to resistance towards the implementation of the digital medication dispenser.

### Coming to trust the digital medication dispenser

The implementation of the medication dispenser included formal training of health professionals, but this had proved difficult due to the working arrangements with staff working at different hours and primarily working out of office. Informal information exchange between professionals was more important, and managers depended on enthusiasts among the staff to motivate colleagues. Defining super users that had key roles during implementation and training was essential for allowing professionals to sharing information and experiences with colleagues, thereby developing their professional practices as a network, not only individually.

Practices had to be developed to ensure which service users that were provided a digital device, and who should have more traditional care arrangements. The team chose who was eligible for a digital dispenser. Individual adaption of care services was the main professional value for these professionals. Standardization did not fit with how they perceived quality of care, nor with their professional values. Not all service users could manage a digital medication dispenser, and safe medication administration had to be maintained. Before trusting service users to utilize the digital device, professionals needed time with the individual user in order to transfer knowledge. This had consequences for their work practices and the organisation of work and care in these services.

*«We can’t push everyone into using the dispenser. It doesn’t suit everybody, and that’s the point. If we are to succeed in implementing welfare technology, we need to adapt it individually. You need to start with the needs of the individual and not start with technology.” (Nurse)*

The greatest concern was if service users had sufficient competence to use the technology. Previous work practices including analog medication devices gave professionals confidence in using digital technology professionally. Through analog devices, they had experienced that technical aids could be beneficial.

*«We’ve had the analogue medication devices for a long time. So. So that was where we learnt to feel safe, to be… move to something with more settings and more complex equipment.» (Nurse)*

However, in their previous care arrangement, the professionals were responsible for service users compliance with their medication regime. With the digital dispenser, the service user him/herself had to take on more of this responsibility. But that would not happen if health professionals themselves lacked trust in the digital solution. Both parties had to be able to interact with the technology, for instance, know what to do when the device started to sound its alarm. Therefore, it was important for professionals that the implementation of the dispenser into a home was well prepared, and that the full care arrangement was thoroughly planned, thereby supporting their willingness to trust the digital medication dispenser to provide safe medication practices.

*«You have to choose carefully where you put this kind of technology. I know people, know how they react and how we need to face them. That is where we should start. We need to allow time before implementing the technology. You can’t present a machine to someone and tell them to push the button – done. We’ll be back on Wednesday.*» *(Nurse).*

As their experiences with the digital medication device grew, these health professionals changed their focus from the technology to focusing on care arrangements. While the set-up of the device was their concern at first, they experienced that it contributed to quality assurance through its functions of digital reporting of aberration from the plan. The new technology led services to rearrange work practices, redefine good care, and reflect on priorities for future home-based care, in which they saw digital technology as part of ensuring quality of care.

*«As time passes, you learn that you need to look at the care arrangement, the design of services. It provides you with new knowledge for the future. If we can work smarter, work in a different manner, we may achieve warmer services, not just care from warm hands.” (Nurse and project leader)*

Through using the digital medication dispenser, these health professionals experienced that care could mean allowing service users to be independent through using technology. Implementing the digital medication dispenser challenged their previous concept of care. Often, health professionals had experienced that it was more efficient to help service users by doing tasks for them rather than training them. In the new understanding, helping was not only doing something for the user but also to allow responsibility and freedom. Thus, care arrangements changed from doing and giving to planning and training service users.

*«I used to think that care is to help them. But as our workdays have become busier, I’ve started to think that they need to do themselves what they are able to do. Because if I do it for you, you might soon enough loose your own ability. So I’ve changed. » (Auxiliary nurse)*

Service users became less dependent on, and less vulnerable to delays in home services. Stories of empowerment and self-management convinced professionals about the usefulness of the digital medication dispenser. Though some service users were troubled by the new technology, for instance by the alarm, others became less dependent on waiting for home services. For some, this meant leaving the house more often, while others could have breakfast in their robe without having professional visitors observing. Thus, the new technology wasn’t just an answer to care needs, but also influenced how professionals interpreted the content of care.

*«Home care services takes up much of a person’s life. Often we are unable to arrive at the planned time, we do have 30 minutes to go on. Potentially, we tie up 3-4 hours of the day for a user – every day! The patient would like to do other stuff than having us visit. Experiencing that dependency is not something many people like. That you’re not able to cope yourself anymore.» (Auxiliary nurse)*

In conclusion, health professionals working in home care services met the policy decision on implementing digital medication dispensers with negotiations on how to use them in practice. For these health professionals, being able to trust that the digital dispenser would provide safe medication practices built on personal experiences with making the technology work for individual patients, taking part in networks with other professionals, and having the opportunity to individualize care arrangements for patients. Moreover, introducing the digital medication dispenser led to professionals needing to take part in more networks to ensure a good care arrangement for each patient.

## Discussion

In the present study, we investigated how health professionals in home care services experienced the implementation process for a digital medication dispenser. These health professionals went through an implementation process where they negotiated the script of the digital medication dispenser to make it fit with arrangements for medication and care that they saw suitable within the frames of professional home care services. First, they negotiated the symbolic meaning of the dispenser, which were meant to make services more effective. Secondly, they took part in reconstructing new care arrangements for patients who were provided a digital medication dispenser. And third, they went through learning processes which allowed them to begin to trust the dispenser to contribute to safe medication practices.

Our study contributes to the claim that it takes more than purchasing technology to make it work in health services [11]. Theorists of science and technology studies suggest that to obtain real implementation of new technology, it must go through a process of domestication [22]. This includes constructing meaning through negotiating symbolic elements, constructing practices for use through negotiating the script, and learning processes of users of the technology [21]. Implementation of digital technology in municipal health care services have previously been met with organizational and technological resistance [13]. A lack of digital competence and infrastructure within the organization could lead to frustration among health professionals in municipal health care services [26]. In the present study, digital technology was perceived as different from familiar technology, which was named technical aids. The digital technology led to a more complex network in which these health professionals had to seek competence when facing technological challenges and their professional work became more dependent on others. Thus, it was not only their individual digital competence that had to develop. How care was arranged also changed.

Policymakers across Europe see digital technology as part of a future sustainable health care service. The construction of problems that the technology is meant to solve will influence how technology is met when implementing it in the care of the elderly [20]. In the present study, we found that health professionals who were working with service users saw different problems for services than those assumed by the decision makers. Health professionals’ concerns was to ensure safe medication practices and good care arrangements for service users, and they opposed symbolic elements of effectivity and innovation. Care is often symbolized by warm hands [10], implying that good care is to be taken care of by another human being. But in the present study, health professionals began to redefine good care after experiencing that service users valued their independence when having a digital medication dispenser.

Experiencing benefits for services users led these health professionals to trust the digital medication dispenser to provide good care. Trust is important in health care since it is vulnerable individuals who are in need of health care services [27]. Therefore, it is important for providers of new technology to manufacture it as trustworthy and safe to use. The digital medication dispensers used in the present study had scripts that included alarms to ensure compliance with taking medication, and locks hindering users to take more medication than prescribed. In this way the script included techniques that minimized future risks, thereby providing elements upon which users may build their trust. But it is not given which elements of knowledge we calculate our trust upon [28, 29]. Moreover, trust could be based on calculations or knowledge, but also on identification [30]. For the health professionals in the present study, calculation of future risks were not sufficient to make them trust the dispenser with medication safety that was their responsibility. Instead of merely following the script of the dispenser, they developed and negotiated practices that allowed trust in specific contexts. One such practice was performing individual evaluation of patients eligible for having a digital medication dispenser. Our results suggest that it is not possible for providers of welfare technology to manufacture trust. In this study trust was gained through identifying the dispenser as a contributor to their professional value of providing good care, thereby being a trustworthy part of the care arrangements of home care services.

Digital technology has been framed as an empowering tool [31]. In the present study, health professionals saw the digital dispenser as empowering only within an individualized care setting. Standardization did not go well with what they defined as good care. Service users in home care services are a diverse group in level of functioning, care needs and digital competence. To be able to tailor care arrangements where the dispenser will contribute to better care, professionals needed resources and the opportunity to exert their professional judgments. Frennert et al. [26] has suggested that implementation of welfare technology can foster processes of standardization, effectivity, and control - traits associated with “McDonaldization”. Professional resistance, as found in the present study, may thus be a buffer against efficiency demands that are inscribed in digital medication dispensers.

### Limitations

Our study was based on five cases in Norway which were among few municipalities that had implemented a digital medication dispenser. This suggests that these cases represent municipalities with an innovative approach. The study participants were recruited to the study by their work manager, and we do not know if other health professionals were less optimistic towards the new welfare technology. A limitation to our study is our choice to see our data through the theoretical perspective of science and technology studies and script [14]. Greenhalgh et al. [32] suggest that it is necessary to include multidisciplinary perspectives in the study of assisted living technology. Using different perspectives could provide a broader understanding of other elements related to the implementation of welfare technology in home care services.

## Conclusions

Implementing digital technology in home care services must be informed by previous practices in the field, especially when it concerns safety for patients. Through processes of domestication, health professionals negotiate technological scripts to make them fit professional ideals and practices. Policymakers and managers must address questions of care arrangements and individualized adaptions to patients’ needs in order to receive support from health professionals when implementing digital technology in home care services. It is not enough to design a working technological devise. Providers of technology and health service managers need to involve health professionals when developing welfare technology. Moreover, the devise needs to be developed within the context where is will be used in order to make sure it fits with real needs, not only with policy. More research implementation of welfare technology in the home care service context is warranted.

## Supplementary information

**Additional file 1.** Interview guides for home health care workers and managers.

## Data Availability

The datasets used and/or analysed during the current study are available from the corresponding author on reasonable request.
