# Supplementary material for: Health professionals’ experiences with the implementation of a digital medication dispenser in home care services – a qualitative study
Source: BMC Health Serv Res. 2020 Apr 16;20:320. doi: 10.1186/s12913-020-05191-9 (PMC7164267; doi:10.1186/s12913-020-05191-9)
Supplement: Supplementary file 1 — Additional file 1. Interview guides for home health care workers and managers. [file 12913_2020_5191_MOESM1_ESM.docx]

**Interview guide for nurses/auxiliary nurses/assistants working in patients’ home**

“*Professional roles and cooperation”*

1. Please describe an ordinary day at work in home care services: what are your tasks and which patients do you see?
2. How would you describe the municipality and home services as a workplace? Specific culture?
3. What are your tasks in helping home dwelling service users with medication? Procedures?
4. How did you conduct medication procedures before implementing the digital technology?

*“The home, and healthcare professionals’ experiences of the technology”*.

1. Please describe how the new digital medication technology works?
2. What must be in place in order for your patients to be able to utilize a digital medication device?
3. In what way does the new technology change your workday?

*“Innovations and change management”*

1. Who are responsible for how the technology works and with whom do you collaborate on the new technology?
2. Do you need new knowledge or competence in order to use the new technology?
3. What are the facilitators and the obstacles to innovation processes in your municipality?
4. What is your personal motivation towards using welfare technology in home care services?
5. Would you say that welfare technology is implemented top-down or bottom-up in your organization?
6. How do you experience the relationship with service users after implementing the new technology? Changed?

**Interview guide for managers/leaders of home care services**

“*Professional roles and cooperation”*

1. How did you conduct medication procedures before implementing the digital technology? (Challenges)?
2. Could you please tell us about the decision process behind choosing the digital dispenser? Was it a top-down or bottom up driven process? Who’s` initiative was it to implement a digital medical dispenser?
3. Please describe how the new digital medication technology works
4. We would like to talk to you about innovation and leading organizational change. Could you please reflect on what you think promote and/or hinders implementation of the electronic medical dispenser in your organization?

*“Innovations and change management”*

1. How did you introduce and organize the implementation of the digital medical dispenser in the organization?
2. Can you please tell us about the collaboration with other partners (such as Research and Development institutions/suppliers) and how this has affected the implementation process?
3. Could you reflect on success factors in the process?
4. Are there particular aspects of medication procedures, for example regulations by law that represent challenges to the implementation process – in someone`s home?
5. In what ways has the new digital dispenser altered the working day for the staff in home care services?
6. We are also interested in the relationship between different professions in the implementation process. Would you be so kind to tell us about the collaboration between different professions in the implementation process this could be possible changes related to responsibility, conflicts, work organization, union participation?
7. How does the medical dispenser affect you as a leader? Do you have experienced need for competence raising in some way?
8. Could you please reflect on how/if the introduction of the digital medical dispenser has exposed new sides of medication to you? (for better or worse)

*“The home, and healthcare professionals’ experiences of the technology”*

1. How do you experience the relationship with service users after implementing the new technology? Changed?
